# Impact of drought and salt stress on galactinol and raffinose family oligosaccharides in common bean (*Phaseolus vulgaris*)

**DOI:** 10.1093/aobpla/plad038

**Published:** 2023-07-05

**Authors:** Ramon de Koning, Gertjan E Wils, Raphaël Kiekens, Luc De Vuyst, Geert Angenon

**Affiliations:** Research Group of Plant Genetics, Faculty of Sciences and Bioengineering Sciences, Vrije Universiteit Brussel, Pleinlaan 2, Brussels, Belgium; Research Group of Plant Genetics, Faculty of Sciences and Bioengineering Sciences, Vrije Universiteit Brussel, Pleinlaan 2, Brussels, Belgium; Research Group of Plant Genetics, Faculty of Sciences and Bioengineering Sciences, Vrije Universiteit Brussel, Pleinlaan 2, Brussels, Belgium; Research Group of Industrial Microbiology and Food Biotechnology, Faculty of Sciences and Bioengineering Sciences, Vrije Universiteit Brussel, Pleinlaan 2, Brussels, Belgium; Research Group of Plant Genetics, Faculty of Sciences and Bioengineering Sciences, Vrije Universiteit Brussel, Pleinlaan 2, Brussels, Belgium

**Keywords:** Abiotic stress, drought, *Fabaceae*, galactinol, RFO, salinity, stress tolerance

## Abstract

Due to climate change, farmers will face more extreme weather conditions and hence will need crops that are better adapted to these challenges. The raffinose family oligosaccharides (RFOs) could play a role in the tolerance of crops towards abiotic stress. To investigate this, we determined for the first time the importance of galactinol and RFOs in the roots and leaves of common bean under drought and salt stress conditions. Initially, the physiological characteristics of common bean under agronomically relevant abiotic stress conditions were investigated by measuring the growth rate, transpiration rate, chlorophyll concentration and membrane stability, allowing to establish relevant sampling points. Subsequently, the differential gene expression profiles of the galactinol and RFO biosynthetic genes and the amount of galactinol and RFO molecules were measured in the primary leaves and roots of *Phaseolus vulgaris* cv. CIAP7247F at these sampling points, using RT-qPCR and HPAEC-PAD, respectively. Under drought stress, the genes *galactinol synthase 1*, *galactinol synthase 3* and *stachyose synthase* were significantly upregulated in the leaves and had a high transcript level in comparison with the other galactinol and RFO biosynthetic genes. This was in accordance with the significantly higher amount of galactinol and raffinose detected in the leaves. Under salt stress, raffinose was also present in a significantly higher quantity in the leaves. In the roots, transcript levels of the RFO biosynthetic genes were generally low and no galactinol, raffinose or stachyose could be detected. These results suggest that in the leaves, both galactinol and raffinose could play a role in the protection of common bean against abiotic stresses. Especially, the isoform galactinol synthase 3 could have a specific role during drought stress and forms an interesting candidate to improve the abiotic stress resistance of common bean or other plant species.

## Teaser

Due to climate change, farmers will face more extreme weather conditions and hence will need crops that are better adapted to these challenges. This study presents new insights into the role of raffinose family oligosaccharides (RFOs) as a resilience mechanism against environmental stress in common bean. This study particularly focuses on galactinol and RFOs in common beans exposed to drought and salt stress conditions. The findings indicate that specific RFO biosynthesis-related genes were significantly upregulated in leaves, coinciding with increased amounts of galactinol and raffinose. No similar patterns were observed in the roots. This points to a potential protective role for galactinol and raffinose in bean leaves against environmental stress. Notably, the isoform galactinol synthase 3 could play a unique role in drought stress resistance, presenting a promising candidate for enhancing climate resilience in common bean or other plant species.

## Introduction

With a growing population to feed and a rapidly changing climate, our agricultural system faces great challenges (Lobell *et al*. 2008). In the transition towards a more sustainable agricultural system, leguminous crops such as common bean (*Phaseolus vulgaris*) are of key importance. The ability of common bean to fix nitrogen through symbiosis with rhizobia makes it less dependent on fertilizers, which reduces the energy consumption of our agricultural system (Herridge *et al*. 2008; Thilakarathna and Raizada 2018; Reinprecht *et al*. 2020). Furthermore, common bean is a great source of nutrients, being rich in carbohydrates, minerals, vitamins, fibre and especially proteins (Doria *et al*. 2012; Campos-Vega *et al*. 2013; Ganesan and Xu 2017). This makes it an excellent alternative source for animal-derived proteins, which are more environmentally costly (Pimentel and Pimentel 2003; Nikbakht Nasrabadi *et al*. 2021). Common bean is the most important grain legume for direct human consumption, especially in Latin America and East Africa, where it is the main source of carbohydrates and proteins in the diets (Broughton *et al*. 2003). In Western countries, however, the cultivation and consumption of common bean still have a large growth potential (Bellucci *et al*. 2021).

As the climate changes, crops will face more extreme weather conditions, such as drought and heat, which also result in increased soil salinity (Mukhopadhyay *et al*. 2021). Currently, around 20 % of the cultivated and 33 % of the irrigated arable land are already affected by salinity, with estimates stating that 50 % of the arable land will be affected by 2050 (Munns and Tester 2008; Ullah *et al*. 2011). Among other crops, common bean is sensitive to saline conditions and even cultivation in slightly saline soil (1 dS m^−1^) will already result in a yield loss of around 20 % (Chinnusamy *et al*. 2005). Furthermore, around 60 % of the dry beans cultivated worldwide are affected by intermittent or terminal drought stress, resulting in severe yield losses (Beebe *et al*. 2013). To tackle these challenges, breeders need in-depth insights into what effects these abiotic stresses have on the plant as well as finding solutions to obtain varieties that are more resilient when exposed to abiotic stress.

Raffinose family oligosaccharides (RFOs) are a family of soluble carbohydrates, derived from sucrose, which contain α-1,6-galactosyl extensions (Peterbauer and Richter 2001). Within common bean, mainly raffinose and stachyose and in lower quantities verbascose can be found in the seeds; however, no quantitative data are available regarding these oligosaccharides in vegetative tissues (Díaz-Batalla *et al*. 2006). The metabolic pathway for the production of RFOs is initiated by the enzyme galactinol synthase, which produces galactinol by the transfer of a galactosyl residue from uridine diphosphate galactose to myo-inositol (Peterbauer and Richter 2001). Galactinol is needed for the formation of the first RFO, raffinose, which is formed by the enzyme raffinose synthase that catalyses the transfer of a galactosyl moiety from galactinol to sucrose (Peterbauer *et al*. 2002a). Stachyose and subsequently verbascose are formed by the transfer of a galactosyl moiety from galactinol to raffinose or stachyose, respectively. Both reactions are catalysed by the stachyose synthase enzyme (Peterbauer *et al*. 2002b). The common bean genome comprises three galactinol synthase genes (*PvGolS1*, *PvGolS2* and *PvGolS3*), two raffinose synthase genes (*PvRS1* and *PvRS2*) and one stachyose synthase gene (*PvSS*; de Koning *et al*. 2021).

In plants, RFOs play a role in the transport and storage of carbon (Sengupta *et al*. 2015). Furthermore, RFOs accumulate during seed maturation to protect them against desiccation and provide seed longevity (Kandler and Hopf 1980; Bailly *et al*. 2001; Blöchl *et al*. 2007). They are also a source of energy and carbon during germination (Peterbauer and Richter 2001; Blöchl *et al*. 2008). Besides these functions under normal plant growth conditions, RFOs and their precursor galactinol could potentially play a role during abiotic stress. Taji *et al*. (2002) have reported that *Arabidopsis thaliana* plants accumulate large amounts of galactinol and raffinose, but no stachyose, under drought, cold and high salinity conditions. In tomato plants, Liu *et al*. (2022) have reported that galactinol synthase is upregulated during cold and heat stress. In chickpea, galactinol synthase is also upregulated during cold, heat, dehydration, salt and oxidative stress (Salvi *et al*. 2018). Furthermore, also the galactinol and raffinose contents are higher under these abiotic stress conditions. In *Coffea arabica*, dos Santos *et al*. (2011) have found that galactinol synthase is upregulated under heat stress, water deficit and high salinity. They have also reported an increase in the accumulation of raffinose and stachyose under these conditions. Under abiotic stress conditions, galactinol and RFOs could help the plant maintain cell turgor by acting as osmolytes, protect the plant by stabilizing proteins and membranes with hydrophilic interactions, and by scavenging reactive oxygen species (ROS) that accumulate in high amounts during these stress conditions (Hincha *et al*. 2003; Seki *et al*. 2007; Nishizawa *et al*. 2008; Peters 2010; Elsayed *et al*. 2014). From an evolutionary point of view, the RFO biosynthesis genes have likely co-evolved with the vascular development of higher plants and could have played an important role in the adaptation of plants from an aquatic to a terrestrial environment, where exposure to drought stress might be common (Yan *et al*. 2022).

In this study, we determined the significance of galactinol and RFOs in common bean during drought and salt stress. Initially, the physiological characteristics of common bean under agronomically relevant abiotic stress conditions were investigated by measuring the growth rate, transpiration rate, chlorophyll concentration and membrane stability, allowing to establish relevant sampling points. Subsequently, the differential gene expression of the galactinol- and RFO synthase genes and the amount of galactinol and RFO molecules were measured in the primary leaves and roots of *P. vulgaris* cv. CIAP7247F at these sampling points using reverse transcription quantitative real-time PCR (RT-qPCR) and high-performance anion-exchange chromatography with pulsed amperometric detection (HPAEC-PAD), respectively.

## Materials and Methods

### Plant material

*Phaseolus vulgaris* cv. CIAP7247F plants were grown in a greenhouse (Brussels, Belgium) under a 16-h light and 8-h dark regime in a mixture of sand and vermiculite (ratio 2:1) in 500-mL perforated pots which were placed on individual trays to prevent water loss. The soil moisture content of all plants was measured daily and the control plants were watered in such a way as to keep the soil moisture content around 80 % of field capacity. To induce drought stress, plants were grown under well-watered conditions until they reached the V3 growth stage (18 days after sowing [DAS]), after which the irrigation was stopped (Vlasova *et al*. 2016). Plants were fertilized with 100 mL of full-strength Hoagland at 14 DAS (Hoagland and Arnon 1950). Leaf and root samples of well-watered control plants and plants in drought stress conditions were harvested and flash frozen with liquid nitrogen at 22 and 25 DAS between 1 p.m. and 2 p.m. to avoid variability due to the circadian rhythm and stored at −80 °C. To induce salt stress, 50 mM NaCl was added to the sand and vermiculite mixture (Chinnusamy *et al*. 2005). Because salt stress causes a reduction in growth rate, plants within this condition were planted 7 days before the control group to make sure the control and salt-stressed plants were in the same developmental stage upon harvesting. Salt-stressed plants were fertilized with 100 mL of full-strength Hoagland at 21 DAS. Samples of the salt-stressed condition were harvested and flash frozen at 29 DAS between 1 p.m. and 2 p.m. and stored at −80 °C.

### Soil moisture content

To determine the soil moisture content at field capacity, the soil was oversaturated with water and covered to prevent evaporation. After draining the soil for 3 consecutive days, the pot containing the drained soil was weighed (*S*_wet_). The soil was then dried in an oven at 110 °C for 24 h to determine the weight of the soil in dry conditions (*S*_dry_). Soil moisture content was calculated using the following formula:


$$\begin{array}{l} Soil\;\;\;moisture\;\;\;content=\;\frac{S_{wet}-S_{dry}}{S_{dry}}\times 100 \end{array}$$ (1.1)

The average of five replicates was used to determine the soil   moisture   content at field capacity. The soil moisture content in pots on a certain day was calculated using Equation (1.1) with *S*_dry_ being the weight of dry soil and pot and *S*_wet_ being the current weight of the soil and pot. The weight of the developing plant was neglected, based on the fact that the average plant weighed only 3 g at growth stadium V3.

The percentage of field capacity of the soil of a certain pot was calculated by dividing that pot’s current soil moisture content by the soil moisture content at field capacity and multiplied by 100 (Equation (1.2)).


$$\frac{\begin{array}{l} {\text{\% Field capacity}}_{\text{pot }i}=\\ {\text{Soil moisture content}}_{\text{pot }i} \end{array}}{{\text{Soil moisture content}}_{\text{at field capacity}}}\times 100$$ (1.2)

### Growth rate

The growth rate was estimated by measuring the area of the primary leaves. The leaf area of five biological replicates was measured using the agronomy tool of APS Assess 2.0 (Lamari 2008). The average and standard error were calculated using Microsoft Excel (v16). IBM SPSS Statistics (v25) was used to perform independent samples *t*-tests to determine the significance of differences between conditions.

### Transpiration rate

The stomatal conductance of the abaxial side of the primary leaves was measured using an SC-1 Leaf Porometer (Decagon Devices, Pullman, WA) around 1 p.m. For each condition, the stomatal conductance was measured of five biological replicates, and the average was calculated using Microsoft Excel (v16). IBM SPSS Statistics (v25) was used to perform independent samples *t*-tests to determine the significance of differences between conditions. Transpiration rates were only compared for measurements taken on the same day because the transpiration rate is highly dependent on environmental conditions and the growth stage of the plant.

### Chlorophyll content

The chlorophyll content of the primary leaves was measured using a SPAD 502 chlorophyll meter 2900P (Spectrum technologies, Bridgend, UK; Richardson *et al*. 2002). For each condition, the SPAD values (numerical value based on the amount of light transmitted by the leaf at 940 and 650 nm) were measured of 5 biological replicates with 10 technical replicates each (measurements randomly spread across the whole leaf) around 1 p.m. and the average was calculated using Microsoft Excel (v16). The chlorophyll content could be derived from the SPAD values using a standard curve (Lichtenthaler and Wellburn 1983; Lichtenthaler 1987). This standard curve was made using leaves with 17 different SPAD values spanning the whole spectrum (from 4.5 to 38.5) and measuring their respective chlorophyll content using a spectrophotometer. To do so, the mid-veins of these 17 leaves were removed and the rest of the leaves were cut into pieces. Of each biological sample, three technical replicates of approximately 100 mg of leaf material were dissolved in 1 mL of 80 % acetone and ground in a mortar. Every sample was then vortexed and incubated in the dark for 2 h during which the samples were gently shaken to extract the chlorophyll. The samples were centrifuged for 60 s at 20 800*g* and the absorbance of the supernatant was measured at 646 and 663 nm with a SmartSpec Plus Spectrophotometer (Bio-Rad, Hercules, CA; Minden *et al*. 2017). The chlorophyll content (µg mg^−1^) of each technical repeat was calculated using the following formula:


$$\frac{\begin{array}{l} \text{Chlorophyll content }\left(\frac{\;\mu \text{ g}}{\text{mg}}\right)=\\ \left(12.21\times E_{663}-2.81\times E_{646}\right)+(20.13\times E_{646}-5.03\\ \times E_{663})\times \text{total volume }\left(\text{mL}\right) \end{array}}{\text{fresh weight }\left(\text{mg}\right)}$$ (1.3)

A graph was made in Microsoft Excel (v16) by plotting the average chlorophyll content of the three technical replicates against the corresponding SPAD value. The standard curve was calculated using exponential regression, which was the best model to fit this dataset (*y* = 0.0967 *e*^0.0736*x*^, *R*^2^ = 0.8811; **see****Supporting Information—Figure S1**; Lichtenthaler and Wellburn 1983; Lichtenthaler 1987; Minden *et al*. 2017).

### Electrolyte leakage

For each condition, three biological replicates were used to measure leaf electrolyte leakage (EL). A primary leaf was cut off at the base of the leaf. To avoid EL, paraffin was applied to the detached surface. Each leaf was gently shaken for 10 min in 100 mL demineralized water inside an individual glass container, closed with a lid to remove the surface solutes. Next, each leaf was placed in a new container containing 100 mL demineralized water and incubated for 2 h in a 35 °C water bath, after which the initial electrical conductivity (EC_1_) of the solution was measured using a SevenGo pro-electrical conductivity meter (Mettler Toledo, Columbus, OH) coupled to an InLab738 conductivity probe (Mettler Toledo). To measure the maximum electrical conductivity (EC_2_), the leaf samples were autoclaved at 121 °C for 20 min to break the cells and release all ions. After the solution was cooled down to 30 °C, the EC_2_ was measured. To calculate the EL, the following formula was used (Dionisio-Sese and Tobita 1998; Kodikara 2018):


$$\begin{array}{l} EL\;=\frac{EC_{1}}{EC_{2}}\;\;\times \;\;100 \end{array}$$ (1.4)

To determine the EL for a certain condition, the average of three biological replicates was calculated using Microsoft Excel (v16). IBM SPSS Statistics (v25) was used to perform independent samples *t*-tests to determine the significance of differences between conditions.

### Differential gene expression of the galactinol- and RFO synthase genes

Frozen root and leaf samples were crushed using the TissueLyser II (Qiagen, Hilden, Germany), and RNA was extracted using the Nucleospin RNA Plant and Fungi kit (Macherey-Nagel, Düren, Germany, CAT #740120.50). The quality and quantity of the samples were checked using the Quantus fluorometer (Promega, Madison, WI), and the RNA integrity was evaluated using a bleach gel electrophoresis, as described by Aranda *et al*. (2012). To remove genomic DNA, the RNA samples were treated with RQ1 RNase-Free DNase (Promega, CAT #M6101), after which cDNA was synthesized using the RevertAid H Minus First Strand cDNA Synthesis Kit (Thermo Fisher Scientific, Waltham, MA, CAT #K1631). Primers used to amplify cDNA were as described by de Koning *et al*. (2021). As a reference gene, *actin-11* (*Act11*) was used, which is stably expressed during abiotic stress conditions (Borges *et al*. 2012). An overview of the primers used can be found in **Supporting Information—Table S1**. To perform RT-qPCR, the samples were mixed with GoTaq qPCR Master Mix (Promega, CAT # A6001) and loaded on the CFX96 Touch Real-Time PCR Detection System (Bio-Rad). The RT-qPCR settings were used as described by de Koning *et al.* (2021). For every condition, the gene expression of the galactinol and RFO biosynthetic genes was measured for three biological replicates, with three technical replicates each. *Actin-11* was used as a reference gene to normalize the cDNA threshold cycle (Ct) values observed by RT-qPCR through Equation (1.5), using the comparative Ct method (Livak and Schmittgen 2001; Schmittgen and Livak 2008; Borges *et al*. 2012):


$$\begin{array}{l} \;\Delta \;Ct_{gene}\;=\;\;Ct_{Act11}-Ct_{gene,\;\;\;mean} \end{array}$$ (1.5)

The difference in gene expression levels was calculated by comparing treatments with the control condition using Equation (1.6) (Livak and Schmittgen 2001).


$$\begin{array}{l} \;\Delta \;\Delta \;Ct_{gene}=\;\Delta \;Ct_{gene,\;mean,\;\;\;treatment}\\ -\;\Delta \;Ct_{gene,\;mean,\;\;\;control} \end{array}$$ (1.6)

IBM SPSS Statistics (v25) was used to perform independent samples *t*-tests to determine the significance of differences in expression.

To calculate the number of gene transcripts of a specific gene relative to the reference gene *Actin-11*, the ∆Ct values of Equation (1.5) were used. During RT-qPCR, the multiplication of cDNA could be calculated with Equation (1.7) (Liu and Saint 2002):


$$\begin{array}{l} N=N_{0}\cdot {\left(1+E\right)}^{n} \end{array}$$ (1.7)

with N: amount of cDNA; $N_{0}:$ start amount of cDNA (or mRNA assuming perfect cDNA synthesis); E: primer pair efficiency; and n: cycle number.

The selected primer pairs used in this analysis had an efficiency between 0.90 and 1.10 (de Koning *et al*. 2021). For further analysis, *E* was approximated by 1. Assuming this, Equation (1.7) could be written as a function of the cycle number (*n*):


$$\begin{array}{l} n=\frac{\ln\left({}^{N}/{}_{N_{0}}\right)}{ln\left(2\right)} \end{array}$$ (1.8)

Equation (1.8) could be substituted in Equation (1.5), resulting in Equation (1.9):


$$\begin{array}{l} \Delta Ct=\frac{\ln\left({}^{N_{Act11}}/{}_{N_{0,Act11}}\right)-\ln\left({}^{N_{gene}}/{}_{N_{0,gene}}\right)}{ln\left(2\right)} \end{array}$$ (1.9)

with *N* equal to the amount of cDNA at the threshold cycle (Ct). At the threshold cycle, the fluorescence signal surpassed a fixed threshold value and the amount of cDNA present (N) was approximately equal for both the reference gene and the other genes $\left(N_{Act11}=\;N_{gene}\right)$, assuming that the primer pairs amplified cDNA fragments of the same length. Using this assumption, Equation (1.9) could be written as


$$\begin{array}{l} ln\left(2\right)\;\Delta \;Ct=\ln\left(\frac{N_{0,gene}}{N_{0,Act11}}\right) \end{array}$$ (1.10)

This could be further written as a ratio between the start amount of cDNA of a specific gene $\left(N_{0,gene}\right)$ and the reference gene $\left(N_{0,Act11}\right)$_:_


$$\begin{array}{l} \frac{N_{0,gene}}{N_{0,Act11}}=e^{ln\left(2\right)\;\Delta \;Ct} \end{array}$$ (1.11)

Equation (1.11) was used to calculate the number of gene transcripts of a specific gene relative to the reference gene *Actin-11.*

### Quantification of the galactinol and RFO content using HPAEC-PAD

Frozen root and leaf samples of around 150 mg were crushed using the TissueLyser II (Qiagen), after which the exact weight of the samples was determined. To each sample, 1 mL 50 % ethanol was added, after which the samples were vortexed and incubated for 30 min at 70 °C, while the samples were gently shaken to enhance the extraction. Next, the samples were centrifuged at 20 800*g* for 15 min, after which the supernatant was filter-sterilized with a 0.2-µm cellulose acetate membrane (VWR, Radnor, PA). One hundred microliters of each sample was added to 900 µL of a solution consisting of 50 % acetonitrile, 49.8 % Milli-Q H_2_O and 0.2 % rhamnose (0.3 mM), which functioned as the internal standard. The samples were vortexed for 2 min and centrifuged at 3600*g* for 15 min, after which the supernatant was filtered through a 0.2 µm Millex-LG Filter (Merck, Darmstadt, Germany) and added to a high-performance liquid chromatography vial.

To quantify the amount of galactinol and RFOs in the samples, high-performance anion-exchange chromatography (HPAEC) with pulsed amperometric detection (PAD) was done, using an ICS 5000 ion chromatography system (Dionex, Sunnyvale, CA) equipped with a Carbopac PA20 column set consisting of a Microbore Guard Column (3 × 60 mm) and a Microbore Analytical Column (150 × 3 mm), which was kept at 35 °C (Kotha *et al*. 2020). For each sample (kept at 10 °C), 10 µL was injected. The mobile phase consisted of Milli-Q water (eluent A), 189 mM NaOH (eluent B) and 1.52 M NaOH (eluent C) and had a flow rate of 0.5 mL min^−1^. To quantify the compounds, the following gradient was applied: 0.0 min, 89 % A and 11 % B; 15.0 min, 100 % B; 21.0 min, 100 % C; 21.5 min, 100 % C; 30.0 min, 89 % A and 11 % B; 35.0 min, 89 % A and 11 % B. The total run time was 35 min.

Stock solutions (50 mg L^−1^) of galactinol (CAS: 16908-86-4, Sigma-Aldrich, St. Louis, MO), raffinose (CAS: 17629-30-0, Sigma-Aldrich) and stachyose (CAS: 54261-98-2, Sigma-Aldrich) were prepared by dissolving each compound in 50 % ethanol, after which 2-fold dilution series were made, ranging from 50 to 0.024 mg L^−1^. Data analysis was done using the Chromeleon Chromatography Data System software (v6.8, Thermo Fisher Scientific), and calibration curves (linearity), limits of detection (LOD) and limits of quantitation (LOQ) were calculated in Microsoft Excel (v16) following the quality guidelines of the International Council for Harmonisation of Technical Requirements for Pharmaceuticals for Human Use (2005). For each condition, the quantities of three biological replicates were measured, and the average values and standard errors were calculated using Microsoft Excel (v16). IBM SPSS Statistics (v25) was used to perform independent samples *t*-tests to determine the quantification significance between conditions. The linearity equations, along with the corresponding LOD and LOQ values, can be found in **Supporting Information—Table S2**.

## Results

### Physiological conditions of *Phaseolus vulgaris* under abiotic stress

**Figure 1.**
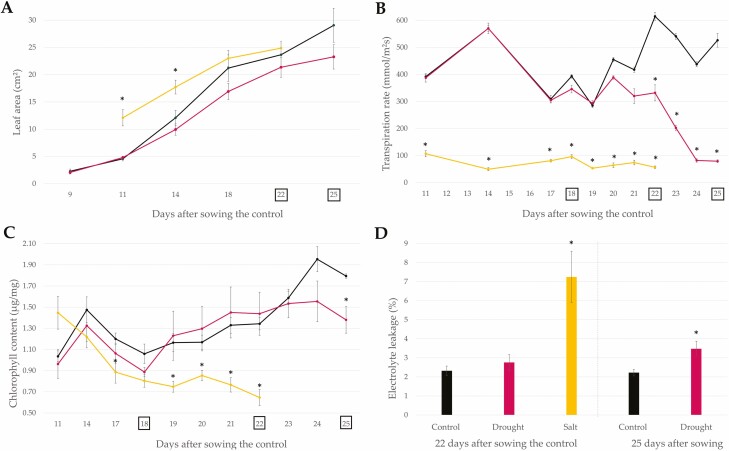
Physiological characteristics of *P. vulgaris* under well-watered (black), drought (pink) and salt (yellow) stress conditions. Salt-stressed plants (50 mM NaCl) were sown 7 days prior to the control plants to compensate for the reduced growth rate. The results for the salt-stressed plants are depicted in terms of the DAS of the control plants. To initiate drought stress, irrigation was stopped at 18 DAS for the drought-stressed plants. (A) Average leaf area of the primary leaves (*n* = 5). (B) Average transpiration rate of the primary leaves (*n* = 5). (C) Average chlorophyll content of the primary leaves (*n* = 5). (D) Average electrolyte leakage in the primary leaves (*n* = 3). Time points with an asterisk (*) above represent values that are significantly different from the leaves of well-watered plants (*P* < 0.05). Error bars represent the standard error.

**Figure 2.**
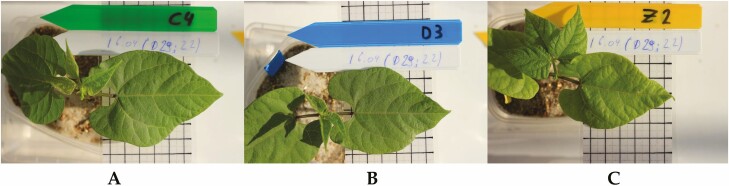
*Phaseolus vulgaris* plants under different abiotic stress conditions in the first trifoliate leaf stage (v3). (A) Control plant 22 DAS (C4). (B) Drought-stressed plant 22 DAS (D3). (C) Salt-stressed plant 29 DAS (Z2).

The growth rate of common bean under well-watered, drought and salt stress conditions was estimated by measuring the leaf area of the primary leaves. All plants reached a similar primary leaf area, with respective values of 23.7 cm^2^ (SE = 2.4) for the control plants 22 DAS, 21.4 cm^2^ (SE = 1.8) for the drought-stressed plants 22 DAS and 24.9 cm^2^ (SE = 1.4) for the salt-stressed plants 29 DAS (Fig. 1A). At this time point, the plants under well-watered, drought and salt stress conditions were also in the same V3 growth stage (Fig. 2; Vlasova *et al*. 2016). The plants under well-watered and drought stress conditions had a similar growth rate, even after the irrigation was stopped 18 DAS (Fig. 1A). For the drought-stressed plants, the soil moisture content dropped from 86.91 % of field capacity (SE = 1.5, *n* = 45) at 18 DAS to 34.2 % (SE = 0.82, *n* = 45) at 22 DAS and reached a value of 17.44 % (SE = 0.43, *n* = 45) at 25 DAS.

The transpiration rate was estimated by measuring the stomatal conductance of the abaxial side of the primary leaves at a fixed time in the day (1 p.m.) once the first true leaves emerged (Fig. 1B). Plants that were subjected to salt stress had a significantly lower transpiration rate in comparison with the control. At 22 DAS, the average stomatal conductance of the salt-stressed plants was 57.0 mmol (m^2^s)^−1^ (SE = 4.7), whereas the control plants had a stomatal conductance of 614.9 mmol (m^2^s)^−1^ (SE = 14.8). After the irrigation was stopped 18 DAS, the first significant difference in stomatal conductance could be seen 22 DAS, where the control plants had an average stomatal conductance of 614.9 mmol (m^2^s)^−1^ (SE = 14.8) as compared to the drought-stressed plants that had an average stomatal conductance of 332.1 mmol (m^2^s)^−1^ (SE = 29.7). The stomatal conductance of the drought-stressed plants dropped in time even further, reaching its lowest point at 25 DAS with an average value of 79.4 (SE = 5.1) mmol (m^2^s)^−1^.

For each condition, the chlorophyll content of the primary leaves was calculated (Fig. 1C). For plants grown under salt stress, the first significant difference in chlorophyll content was observed at 17 DAS for the control (24 DAS for the salt-stressed plants), with the salt-stressed plants having an average chlorophyll content of 0.89 μg mg^−1^ (SE = 0.10) in comparison with the control that had an average chlorophyll content of 1.20 μg mg^−1^ (SE = 0.06). The chlorophyll content of the salt-stressed plants dropped over time, reaching its lowest value at 22 DAS the control, with an average value of 0.65 μg mg^−1^ (SE = 0.08). Plants under drought stress and control conditions had a similar chlorophyll content, except at 25 DAS, at which the drought-stressed plants had an average chlorophyll content of 1.38 μg mg^−1^ (SE = 0.13), which was significantly lower than the control plants (1.79 μg mg^−1^; SE = 0.02).

The membrane stability was estimated by measuring the EL in the primary leaves (Fig. 1D). Plants grown under salt stress conditions showed a significant increase in EL in comparison to the control plants 22 days after sowing the control, with an average EL of 7.25 % (SE = 1.35) in comparison with the control that had an average EL of 2.32 % (SE = 0.25). Plants grown under drought stress conditions only showed a significant increase in EL 25 DAS, with an average EL of 3.48 % (SE = 0.40) in comparison with the control that had an average EL of 2.22 % (SE = 0.17).

### Differential gene expression of the galactinol and RFO synthase genes under abiotic stress conditions

For every condition, the gene expression of the galactinol and RFO biosynthetic genes was measured in the primary leaves and roots of *P. vulgaris* using RT-qPCR. The differential gene expression is represented as ΔΔCt value, of which positive values represent an upregulation and negative values a downregulation of the specific gene in comparison with the control plants.  Δ Δ Ct values close to zero indicate no substantial change in gene expression in comparison to the control condition (Livak and Schmittgen 2001; Schmittgen and Livak 2008).

**Figure 3.**
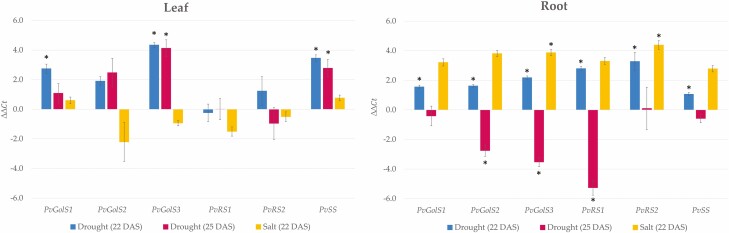
Differential gene expression of the galactinol and RFO synthase genes in the primary leaves and roots of *P. vulgaris* under an early stage of drought stress (blue), more severe drought stress (red) and salt stress conditions (yellow). The differences in gene expression levels of *galactinol synthase 1* (*PvGolS1*), *galactinol synthase 2* (*PvGolS2*), *galactinol synthase 3* (*PvGolS3*), *raffinose synthase 1* (*PvRS1*), *raffinose synthase 2* (*PvRS2*) and *stachyose synthase* (*PvSS*) are represented by  Δ Δ Ct values. Bars with an asterisk (*) above represent values that are significantly different from the control samples of well-watered plants (*P* < 0.05) and error bars represent the standard error.

Under drought stress, common bean plants showed overexpression of *galactinol synthase 1* (*PvGolS1*), *galactinol synthase 3* (*PvGolS3*) and *stachyose synthase* (*PvSS*) in the primary leaves in comparison with the control (Fig. 3). During the early stages of drought stress (22 DAS), *PvGolS1* was significantly upregulated having a  Δ Δ Ct value of 2.8 (SE = 0.3). Both *PvGolS3* and *PvSS* were also significantly upregulated at this stage with respective  Δ Δ Ct values of 4.4 (SE = 0.2) and 3.5 (SE = 0.2). Under more severe drought stress (25 DAS), *PvGolS3* and *PvSS* were still significantly upregulated with respective  Δ Δ Ct values of 4.1 (SE = 0.6) and 2.8 (SE = 0.6). When looking at the primary leaves of salt-stressed plants, no significant difference in the expression of the galactinol and RFO synthase genes was observed, demonstrated by  Δ Δ Ct values close to zero.

In the roots, the differential gene expression of the galactinol and RFO synthase genes was more complex (Fig. 3). During the early drought stress stage (22 DAS), all galactinol and RFO synthase genes showed a significant upregulation. In contrast, under more severe drought stress (25 DAS), *galactinol synthase 2* (*PvGolS2*), *PvGolS3* and *raffinose synthase 1* (*PvRS1*) were significantly downregulated, having respective ΔΔCt values of −2.8 (SE = 0.4), −3.5 (SE = 0.3) and −5.3 (SE = 0.5). Under salt stress, *PvGolS3* and *raffinose synthase 2* (*PvRS2*) were significantly upregulated, with respective  Δ Δ Ct values of 3.9 (SE = 0.2) and 4.4 (SE = 0.3).

**Figure 4.**
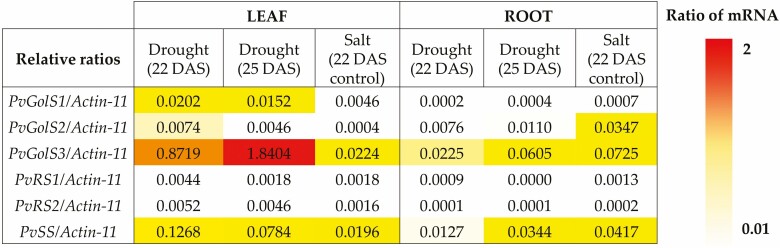
Heat map of the estimated number of gene transcripts of the galactinol and RFO synthase genes relative to the gene transcripts of the reference gene (*Actin-11*) in the leaves and roots of *P. vulgaris* during different abiotic stress conditions. The relative number of gene transcripts is represented by a colour gradient, in which the white colour indicates a low ratio (<0.01) and the red colour a high ratio (>2).

Besides the measurements of differential gene expression, the estimated number of gene transcripts of the galactinol and RFO synthase genes relative to the reference gene *Actin-11* was calculated (Fig. 4). In the primary leaves of drought-stressed plants, the genes *PvGolS1*, *PvGolS3* and *PvSS* showed a high relative number of gene transcripts in comparison with the other galactinol and RFO synthase genes. Especially *PvGolS3*, with values of 0.8719 and 1.8404, respectively, during the early stage of drought stress (22 DAS) and at the more severe stage (25 DAS). *PvSS* came second with values of 0.1268 at 22 DAS and 0.0784 at 25 DAS, and *PvGolS1* had a relative number of gene transcripts of 0.0202 and 0.0152 at 22 and 25 DAS, respectively. Under salt stress conditions, most galactinol and RFO synthase genes had a very low relative number of gene transcripts in the primary leaves, with only *PvGolS3* and *PvSS* having slightly higher numbers (0.0224 and 0.0196, respectively). In the roots, the relative number of gene transcripts for all galactinol and RFO synthase genes was much lower compared to the leaves. Although still low, *PvGolS3* and *PvSS* had slightly higher values in the roots in comparison with the other galactinol and RFO synthase genes.

### Quantification of the galactinol and RFO content under abiotic stress conditions

**Figure 5.**
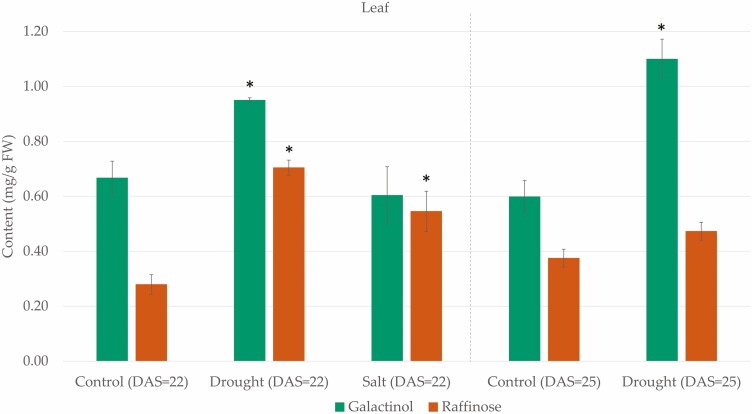
Quantification of the galactinol (green) and raffinose (orange) content in the primary leaves of *P. vulgaris* under abiotic stress conditions using HPAEC-PAD. The average content per gram of fresh weight (FW) is shown for three biological replicates and the error bars represent the standard error. Bars with an asterisk (*) above represent values that are significantly different from the well-watered control plant samples (*P* < 0.05).

The galactinol and RFO content were measured in the primary leaves and roots of *P. vulgaris* under well-watered, drought and salt stress conditions using HPAEC-PAD. Under drought stress, the galactinol and raffinose content in the primary leaves both significantly increased in comparison with the control (Fig. 5). During the early stage of drought stress (22 DAS), the primary leaves of the drought-stressed plants contained 42 % more galactinol than the control plants. They contained on average 0.95 mg galactinol per g fresh weight (FW; SE = 0.01) in comparison to 0.67 mg galactinol per g FW (SE = 0.06) in the control plants. The raffinose content also significantly increased by 150 % in the primary leaves at this stage, containing on average 0.70 mg raffinose per g FW (SE = 0.03), whereas the control plants contained on average 0.28 mg raffinose per g FW (SE = 0.04). Under more severe drought stress (25 DAS), the galactinol content increased by 83 %, reaching a content of 1.10 mg galactinol per g FW (SE = 0.07) in the primary leaves, while the control plants only contained 0.60 mg galactinol per g FW (SE = 0.06). At this stage, the raffinose content also increased although not significantly. During salt stress, only a significant increase in the raffinose content was observed in the primary leaves. The salt-stressed plants contained 96 % more raffinose than the control, reaching a value of 0.55 mg raffinose per g FW (SE = 0.07) in comparison to 0.28 mg raffinose per g FW (SE = 0.04) in the control plants. In the roots, no galactinol, raffinose nor stachyose was measured above the LOD values in any condition.

## Discussion

This study investigated the potential role that galactinol and RFOs play in the tolerance mechanism of common bean under agronomically relevant abiotic stress conditions. Gene expression and metabolite accumulation were examined in drought and salt stress conditions, making sure to compare plants in the same growth stage for accurate evaluation (Farooq *et al*. 2017). Moreover, the physiological characteristics of plants under stress were carefully monitored to establish relevant sampling points. During drought stress, the first physiological signs appeared 4 days after the irrigation was stopped, at 22 DAS, where for the first time, the stomatal conductance was significantly lower than for the well-watered control plants (Fig. 1B). The closure of stomata is considered an early sign of drought stress, reducing the water loss of the plant by lowering the transpiration rate (Farooq *et al*. 2017). This, however, also results in a reduction of carbon influx, lowering the internal concentration of CO_2_ in the leaves, which is considered the main limiting factor for photosynthesis during drought stress and as a consequence reduces the production of saccharides needed for plant growth (Chaves 1991; Cornic 2000; Flexas *et al*. 2004; Farooq *et al*. 2017). At 25 DAS, the first signs of more severe stress were observed. At this time point, the chlorophyll content in the primary leaves was significantly lower than the control and the EL was significantly higher(Fig. 1C and D). During abiotic stress, ROS are being formed, which initially function as signalling molecules, triggering defence mechanisms (Foyer and Noctor 2005). However, during more severe or prolonged stress, the concentration of ROS can further increase and damage the chloroplasts, reducing the chlorophyll content (Mafakheri *et al*. 2010). Furthermore, ROS can oxidize proteins and membrane lipids, which destabilizes the membrane and leads to EL (Gaspar *et al*. 2002). Both of these sampling points are indicative of the plant’s response to drought stress at an early (22 DAS) and more severe stage (25 DAS) and are good sample points to investigate the role of galactinol and RFOs. To induce agronomically relevant salt stress conditions, plants were grown from the start in saline soil of 50 mM NaCl, which corresponds with an electrical conductivity of the soil of approximately 5 dS m^−1^. By definition, soils are considered saline if they have an electrical conductivity of 4 dS m^−1^ or more (Chinnusamy *et al*. 2005). Twenty-two days after sowing the control, the plants were in the same V3 growth stage as the control plants (Fig. 2). At this time point, the plants experienced salt stress, as the transpiration rate was significantly lower than for the control plants (Fig. 1B). Like drought stress, plants subjected to salt stress experience osmotic stress and try to limit their water loss by closing the stomata. On top of the osmotic stress, salt-stressed plants also experience ionic stress caused by the uptake of high amounts of Na^+^ and Cl^-^ ions (Tavakkoli *et al*. 2010). These stresses can cause a decrease in chlorophyll content and an increase in EL, which corresponded with the measurements in the salt-stressed plants of the present study (Fig. 1C and D; Tavakkoli *et al*. 2010). Both of these physiological traits indicate that the plants experienced more severe salt stress and demonstrate that this sample point was suitable to determine the role of galactinol and RFOs during long-term salt stress exposure (Gaspar *et al*. 2002; Flexas *et al*. 2004; Chinnusamy *et al*. 2005; Demidchik *et al*. 2014).

Previous studies have shown that there is a relationship between abiotic stress and an increase of galactinol and RFOs in plants and that these components could help the plant mitigate these stresses (Hincha *et al*. 2003; Seki *et al*. 2007; Nishizawa *et al*. 2008; Peters 2010; Elsayed *et al*. 2014). However, this has not been investigated in common bean so far. To understand the significance of these saccharides, we quantified galactinol and RFOs in leaves and roots and measured the expression of their biosynthetic genes. While interpreting the up- or downregulation of a gene, it is important to also take into account the number of gene transcripts present. A doubling of the gene expression may not be relevant if the initial amount of gene transcripts is very low. To estimate this amount, the number of gene transcripts of the galactinol and RFO synthase genes were calculated relative to the reference gene *Actin-11* (Borges *et al*. 2012).

During early drought stress (22 DAS), the galactinol synthase genes *PvGolS1* and *PvGolS3* and the stachyose synthase gene *PvSS* were significantly upregulated in the primary leaves of common bean (Fig. 3). During more severe drought stress (25 DAS), *PvGolS3* and *PvSS* were still significantly upregulated. Besides their upregulation, these genes also had a high relative number of gene transcripts present (Fig. 4). Especially *PvGolS3* showed high values at both the early and more severe drought stress conditions. These results correspond to the significantly higher amount of galactinol observed in the primary leaves during both drought stress stages (Fig. 5). These results highlight the potential importance of isoform *PvGolS3* in producing galactinol during drought stress in the leaves of common bean. Raffinose was also present in a significantly higher amount during the early stage of drought stress and slightly higher at the more severe stage. However, no overexpression of raffinose synthase was observed. It could be that due to the high concentration of galactinol, raffinose synthase can produce significantly more raffinose without the need for higher quantities of the enzyme (Vinson *et al*. 2020). Because *PvSS* showed an overexpression at both stages, we would have expected to see an increase in stachyose as well; however, no stachyose was detected. Stachyose synthase can produce, besides stachyose, also galactosyl cyclitols that have a similar chemical structure as RFOs and could potentially also provide tolerance towards abiotic stress (Obendorf 1997; Frias *et al*. 1999). Overexpression of *PvSS* might increase the production of these galactosyl cyclitols; however, the concentration of the galactosyl cyclitols was not measured in this study. The concentrations of galactinol and RFOs measured in the leaves of common bean were in correspondence with the concentrations found in the leaves of other plant species, such as *Cucumis sativus* and *A. thaliana* under abiotic stress (Sun *et al*. 2013; Gu *et al*. 2018). The roots of common bean under drought stress showed a contrasting expression pattern for the galactinol synthase genes. These genes were upregulated during early drought stress (22 DAS) but showed a downregulation at the more severe stage (25 DAS; Fig. 3). The same contrasting pattern was observed for the raffinose synthase gene, *PvRS1*. The genes *PvRS2* and *PvSS* only showed a significant upregulation during early drought stress. However, these differential gene expression patterns could be less relevant because the estimated number of gene transcripts present for all these genes was very low (Fig. 4). Moreover, no galactinol nor RFOs were detected in the roots, indicating that these saccharides are either not produced, or rapidly broken down in the roots (Eom *et al*. 2012). The low relative abundance of gene transcripts of the RFO synthase genes further indicates that RFOs are not produced in the roots. Our results are in correspondence with previous research on common bean and on homologous genes found in other species. In common bean, an increase in raffinose was also detected in the stem and seed under drought stress conditions (Andrade *et al*. 2016; Herrera *et al*. 2019). Taji *et al*. (2002) have discovered in *A. thaliana* that stress-inducible galactinol synthase genes play an important role in the accumulation of galactinol and raffinose under drought stress conditions and could provide drought tolerance. Selvaraj *et al*. (2017) have shown that overexpression of such stress-inducible galactinol synthase gene (*AtGolS2*) of *A. thaliana* in transgenic rice results in plants with an elevated galactinol content in the leaves, which improves drought tolerance and grain yield in drought stress conditions. They have observed better water retention, higher photosynthesis activity and faster recovery in the overexpressing line during drought stress, which could be related to the elevated galactinol content. In *C. sativus*, Ma *et al*. (2021) have shown that overexpression of a galactinol synthase gene (*CsGolS4*) leads to a higher amount of galactinol and RFOs and reduced levels of ROS in the leaves, demonstrating a higher tolerance against drought and cold stress. Vinson *et al*. (2020) have demonstrated an increase in the drought and salt stress tolerance of *A. thaliana* plants overexpressing a galactinol synthase gene (*AdGolS3*) of *Arachis duranensis.* These plants had a significantly higher concentration of galactinol and raffinose in the leaves during abiotic stress, which results in better water retention, membrane stability and protection against oxidative stress.

Under salt stress, no significant up- or downregulation of the galactinol or RFO synthase genes could be seen in the primary leaves of common bean (Fig. 3). In the roots, the galactinol synthase gene *PvGolS3* and the raffinose synthase gene *PvRS2* were significantly upregulated (Fig. 3). However, the estimated transcript abundance of these genes was very low, questioning the significance of these upregulations (Fig. 4). Furthermore, no galactinol nor RFOs were detected in the roots. In the primary leaves, however, a significantly higher raffinose content was measured, which may indicate a possible role of raffinose in common bean plants under prolonged salt stress (Fig. 5). In other species, such as *A. thaliana* and *C. arabica*, an increase in the raffinose content together with an increase in the galactinol or stachyose content, respectively, has also been reported in plants subjected to salt stress (Taji *et al*. 2002; dos Santos *et al*. 2011). Sun *et al*. (2013) have discovered that the overexpression of a galactinol synthase gene (*TsGolS2*) from *Thellungiella salsuginea* in *A. thaliana* plants increases the amount of galactinol and raffinose in the leaves after exposure to high salinity and osmotic stress and improves the tolerance of the plants against these stresses.

During abiotic stress conditions, galactinol and RFOs could have multiple functions providing protection against osmotic and ionic stress. First, they can maintain cell turgor by acting as osmolytes (Bartels and Ramanjulu 2005). Second, they can stabilize proteins and membranes through hydrophilic interactions, ensuring normal protein functioning and preventing cell leakage (Hincha *et al*. 2003). Moreover, research has demonstrated that raffinose can be transported to the chloroplasts, where it can protect the thylakoids and stabilize photosystem II (Schneider and Keller 2009; Knaupp *et al*. 2011). Last, galactinol and RFOs can scavenge ROS, which could otherwise damage the plant (Nishizawa *et al*. 2008). Galactinol and raffinose have a similar capacity to scavenge ROS (Nishizawa *et al*. 2008). However, galactinol is energy-wise a better osmolyte than raffinose and stachyose, because only the concentration of osmolytes is important to create osmotic pressure and not the chemical properties of the solute (Seki *et al*. 2007). Therefore, the production of a certain concentration of solutes is less energy demanding when the plant accumulates galactinol and sucrose compared to the accumulation of raffinose or stachyose, which are trisaccharides and tetrasaccharides, respectively. From this, it could be hypothesized that the overproduction of galactinol would be the better option to provide abiotic stress resistance in plants. Besides the function of galactinol and RFOs in protecting against abiotic stress, they can also aid in plant recovery after plants underwent abiotic stress by serving as an energy source (Peters *et al*. 2007). In common bean or other plant species, the isoform *PvGolS3* could be a promising candidate to improve the tolerance towards abiotic stress. The overexpression of this gene by inserting an extra copy of it in the genome could lead to higher levels of galactinol and raffinose under abiotic stress conditions protecting the plant. Previous research has shown that under normal plant growth conditions, *PvGolS3* is mainly expressed in the vegetative tissue of common bean and no expression has been found in the seeds (de Koning *et al*. 2021). Therefore, an extra copy of it in the genome would not contribute to an increased amount of RFOs in the seed, because RFOs are produced *de novo* during seed maturation (Peterbauer and Richter 2001). Within the seeds, RFOs are among the most important anti-nutritional factors, causing flatulence and digestive problems when consumed by humans and monogastric animals, so an increase in the concentration in the seed should be avoided (Tomlin *et al*. 1991; Yamaguishi *et al*. 2009; Doria *et al*. 2012; Valentine *et al*. 2017).

## Supporting Information

The following additional information is available in the online version of this article –

**Figure S1**. Relationship between the chlorophyll content of the leaves of 17 *P. vulgaris* cv. CIAP7247F plants and their corresponding SPAD values.

**Table S1**. Primers used for RT-qPCR.

**Table S2**. Analysis of the galactinol and raffinose family oligosaccharides content in root and leaf samples of *P. vulgaris* CIAP7247F under different abiotic stress conditions using HPAEC-PAD.

plad038_suppl_Supplementary_Material

## Data Availability

The datasets generated and analysed during the current study are incorporated in the manuscript and its supplementary materials. These include the tables, figures and additional data used to support the findings of this study. If further information is needed, please contact the corresponding author.

## Notes

**Phenome, Genome and Environment. Chief Editor:** Colleen Doherty
