# Supplementary material for: Impact of drought and salt stress on galactinol and raffinose family oligosaccharides in common bean (Phaseolus vulgaris)
Source: AoB Plants. 2023 Jul 5;15(4):plad038. doi: 10.1093/aobpla/plad038 (PMC10327629; doi:10.1093/aobpla/plad038)
Supplement: plad038_suppl_Supplementary_Material [file plad038_suppl_supplementary_material.pdf]

Supporting Information

Impact of drought and salt stress on galactinol and raffinose family oligosaccharides in common bean (*Phaseolus vulgaris* L.)

Ramon de Koning, Gertjan E. Wils , Raphaël Kiekens, Luc De Vuyst, Geert Angenon

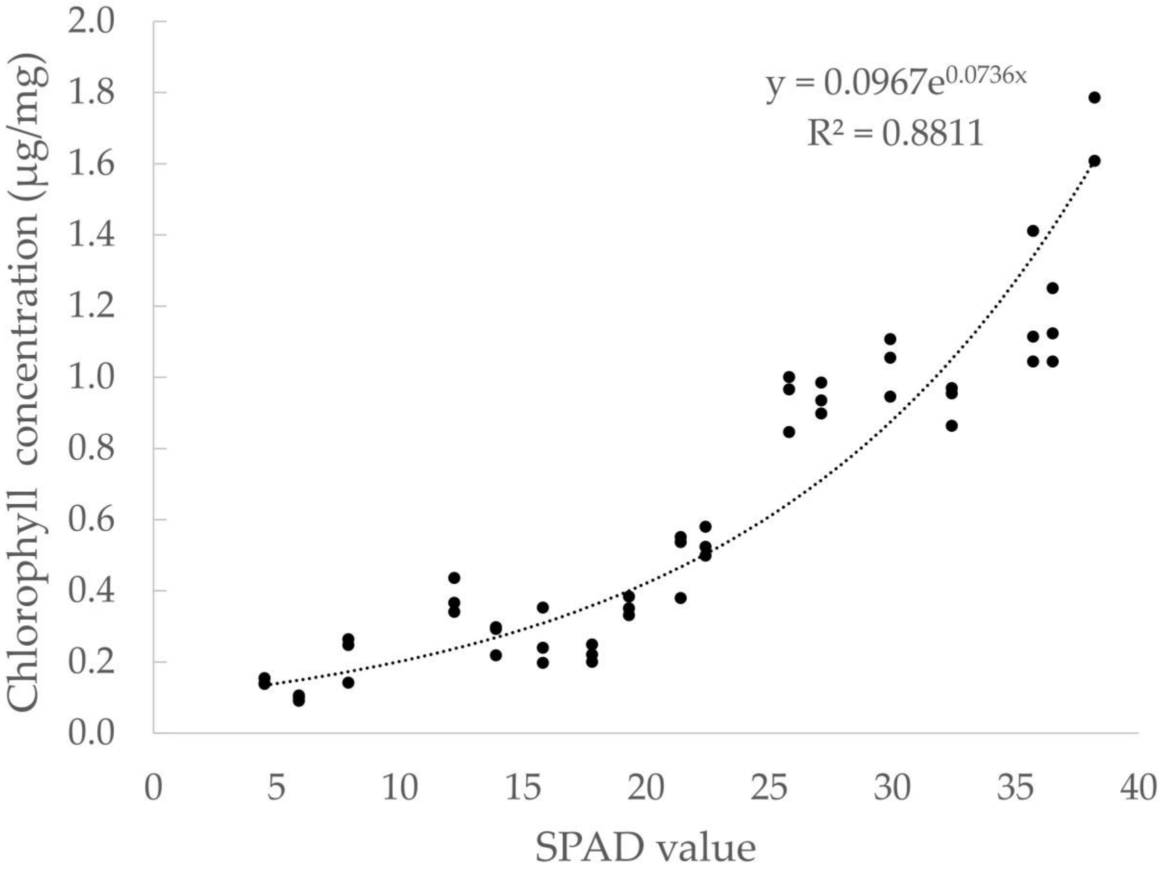

Figure S1. Relationship between the chlorophyll content of the leaves of 17 *P. vulgaris* cv. CIAP7247F plants and their corresponding SPAD values (ranging from 4.5 to 38.2). The leaf chlorophyll content of each plant is represented by three technical replicates.

Table S1. Primers used for RT-qPCR.

*Accession numbers were adopted from Phytozome v12.1 (*P. vulgaris* v2.1).

**Accession Number***	**Gene name**	**Forward Primer**	**Tm (°C)**	**Reverse Primer**	**Tm (°C)**	**Amplicon length (nt)**
Phvul.001G215300	*PvGolS1*	$^{5'}$CGCGTAGGAG ATGACTTTTG$^{3'}$	60.5	$^{5'}$GCACATGGG TGAGTAGATTG$^{3'}$	60.4	103
Phvul.001G223700	*PvGolS2*	$^{5'}$ACAGTGCCCTA ATAAAGTTCAG$^{3'}$	60.4	$^{5'}$GTTCAGAAA GTCCTGCTCAG$^{3'}$	60.2	168
Phvul.007G203400	*PvGolS3*	$^{5'}$GAATTTGGGA GTTTGTGGAG$^{3'}$	58.9	$^{5'}$AGAAGTAGTTAT CAGGCAAGTC$^{3'}$	59.6	161
Phvul.004G007100	*PvRS1*	$^{5'}$GACAATATTT CGGTGTGCGT$^{3'}$	61	$^{5'}$TAGAAGGTTGA AGCTTCCCA $^{3'}$	60.9	155
Phvul.009G175400	*PvRS2*	$^{5'}$GACCCATTTAC GTTAGTGATTGTG$^{3'}$	61.8	$^{5'}$CACCTGTATGTT TGTTGAGATTCC$^{3'}$	61.8	131
Phvul.001G214300	*PvSS*	$^{5'}$CTCATTAAGAAG CTTGTCTTCCC$^{3'}$	61.1	$^{5'}$CTCCTCCATACT TATTGAAGTTCC$^{3'}$	60.5	144
Actin-11 (Borges *et al.*, 2012)	*Actin-11*	$^{5'}$TGCATACGTT GGTGATGAGG$^{3'}$	62.2	$^{5'}$AGCCTTGGGGTT AAGAGGAG$^{3'}$	63.2	190

Table S2. Analysis of the galactinol and raffinose family oligosaccharides content in root and leaf samples of *P. vulgaris* CIAP7247F under different abiotic stress conditions using HPAEC-PAD. The blue colour indicates values above the limits of quantification (LOQ) whereas the red colour indicates values below the limits of detection (LOD).

	Linearity (mg/l)	Equation	R^2	LOD (mg/l)	LOQ (mg/l)
Galactinol	0.024 - 50	y = 0.0329x + 0.0008	0.9971	0.84	2.55
Raffinose	0.024 - 50	y = 0.0332x - 0.0071	0.9997	0.24	0.73
Stachyose	0.024 - 50	y = 0.063x + 0.0099	0.9996	0.29	0.89
	**Leaf content in HPLC vial (n = 3, mg/l)**				
	Control (DAS = 22)	Drought (DAS = 22)	Salt (DAS = 22)	Control (DAS = 25)	Drought (DAS = 25)
Galactinol	10.49 ± 0.1.67	11.91 ± 0.41	7.76 ± 1.67	8.15 ± 0.77	14.34 ±1.36
Raffinose	4.31 ± 0.59	8.84 ± 0.57	6.8 ± 0.65	5.13 ± 0.54	6.16 ± 0.54
Stachyose	-	0.15 ± 0.05	0.14 ± 0.14	-	-
	**Root content in HPLC vial (n = 3, mg/l)**				
	Control (DAS = 22)	Drought (DAS = 22)	Salt (DAS = 22)	Control (DAS = 25)	Drought (DAS = 25)
Galactinol	0.27 ± 0.16	0.66 ± 0.19	0.37 ± 0.09	0.53 ± 0.33	0.52 ± 0.16
Raffinose	-	-	0.09 ± 0.09	-	0.18 ± 0.18
Stachyose	-	-	-	-	-
